# Oblique Axis Body Fracture: An Unstable Subtype of Anderson Type III Odontoid Fractures—Apropos of Two Cases

**DOI:** 10.1155/2016/7561682

**Published:** 2016-03-06

**Authors:** Hirokazu Takai, Lukas Konstantinidis, Hagen Schmal, Peter Helwig, Stefan Knöller, Norbert Südkamp, Oliver Hauschild

**Affiliations:** ^1^Department of Orthopaedic Surgery and Traumatology, Freiburg University Medical Centre, 79106 Freiburg im Breisgau, Germany; ^2^Department of Orthopaedics and Traumatology, Odense University Hospital, Sonder Boulevard 29, 5000 Odense C, Denmark

## Abstract

*Purpose*. Anderson type III odontoid fractures have traditionally been considered stable and treated conservatively. However, unstable cases with unfavorable results following conservative treatment have been reported.* Methods*. We present the cases of two patients who sustained minimally displaced Anderson type III fractures with a characteristic fracture pattern that we refer to as “oblique type axis body fracture.”* Results*. The female patients aged 90 and 72 years, respectively, were both diagnosed with minimally displaced Anderson type III fractures. Both fractures had a characteristic “oblique type” fracture pattern. The first patient was treated conservatively with cervical spine immobilization in a semirigid collar. However, gross displacement was noted at the 6-week follow-up visit. The second patient was therefore treated operatively by C1–C3/4 posterior fusion and the course was uneventful.* Conclusions*. Oblique type axis body fractures resemble a highly unstable subtype of Anderson type III fractures with the potential of severe secondary deformity following conservative treatment, irrespective of initial grade of displacement. The authors therefore warrant a high index of suspicion for this injury and suggest early operative stabilization.

## 1. Introduction

One of the most widely accepted classifications for odontoid fractures is that of Anderson and D'Alonzo. According to the classification type III fractures extend downward into the cancellous portion of the axis body [1]. Type III fractures are generally considered stable and traditionally conservative treatment has been recommended [2, 3]. However, cases of unstable fractures associated with poor clinical outcome following conservative treatment have been described [4, 5]. Unacceptably high nonunion rates have been reported for type III odontoid fractures in case of severe displacement or so-called shallow-type fractures following conservative treatment. Thus, operative treatment has been favored in these cases [6–8].

For an optimal choice of treatment modality it is crucial that we identify unstable Anderson type III fractures. In this series, we describe two cases of an unstable Anderson III fracture subtype which fit neither displaced fractures nor “shallow-type” fractures. The subtype is referred to as “oblique axis body type” fracture.

## 2. Case Presentation

### 2.1. Case 1

**Figure 1:**
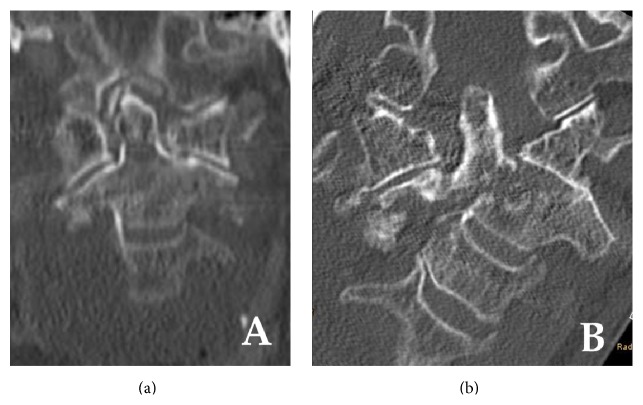
Coronal plane cervical spine computed tomography (CT) images of a 90-year-old woman. (a) Initial imaging showing minimal displacement and good cancellous bony contact and the typical oblique fracture pattern. (b) Follow-up imaging after 6 weeks of collar immobilization showing severe lateral displacement and tilting with dislocation of the left C1/2 facet joint.

A ninety-year-old lady with dementia was referred following a fall when mobilized to her wheelchair at a nursing home for the aged. The patient had been suffering from severe dementia and was immobile for several years prior to the injury. She presented with pain in her neck and left hip. Glasgow Coma Scale (GCS) was 15. There was no neurological disturbance. Radiographs and computed tomography (CT) showed a minimally displaced Anderson type III odontoid fracture (Figure 1(a)) and cut-out of a proximal femoral nail implanted several months earlier for a trochanteric fracture. With regard to her limited level of activity implant removal and primary Girdlestone resection were performed on the left hip. The odontoid fracture was treated conservatively and a semirigid Miami-J collar was applied. She had severe dementia so that the collar was worn by her family and the staff of the nursing home. The doctor had indicated the clinic revisit after 2 weeks, but the family of the patient did not bring the patient to the hospital as she had no pain. After 6 weeks, the patient was taken to the hospital because of the severe deformity of her neck. Computed tomography scans showed a severe displacement of the odontoid fracture (Figure 1(b)). Fortunately, the patient reported no pain and neurologic exams showed no sensomotoric deficits. Posterior fusion was discussed but the patient and her relatives refused and opted for a wait-and-see strategy with permanent cervical immobilization.

### 2.2. Case 2

**Figure 2:**
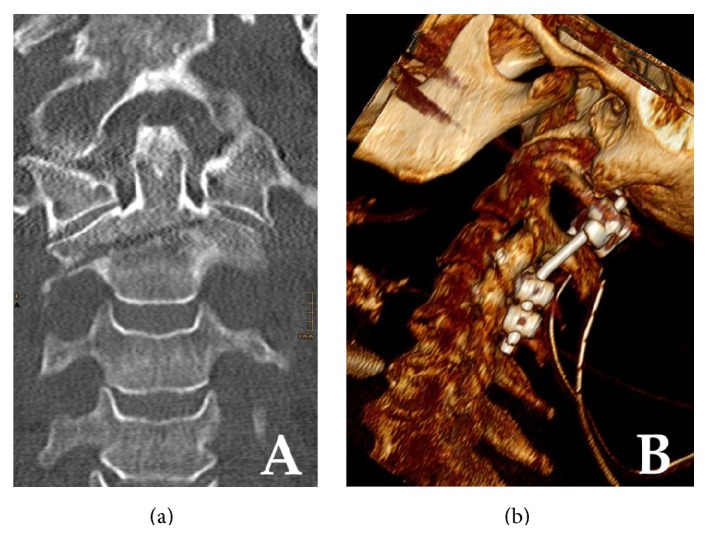
Cervical spine CT imaging of a 71-year-old woman. (a) Coronal plane CT imaging showing the minimally displaced oblique type axis body fracture. Note the marked similarity of the fracture pattern when compared to that shown in Figure 1(a). (b) Three-dimensional computed tomographic VRT reconstruction following C1–C3/C4 posterior fusion with a polyaxial screw-rod construct.

A seventy-two-year-old polytraumatized woman was referred after a high velocity motor vehicle accident. On admission the morbidly obese case (body mass index 52 kg/m^2^) was hemodynamically stable with mild respiratory distress; there was no neurologic deficit. Whole body multislice CT was performed and she was diagnosed with cerebral concussion, blunt thoracic trauma with left clavicle fracture, bilateral rib fractures, fracture and pulmonary contusion, a minimally displaced Anderson type III odontoid fracture and C7 spinal process fracture, AO type B1 right distal radius fracture, left hip dislocation fracture (Pipkin type II), and left Weber/Danis type C ankle fracture (ISS: 29). After cast immobilization of the right forearm and left ankle and application of a semirigid Miami-J collar, primary hip arthroplasty was performed for the hip dislocation fracture. The distal radius and ankle fractures were treated by open reduction and internal fixation after initial cast immobilization several days later. We recognized that there was a marked similarity of the axis body fracture with that described in case 1. Bearing the severe secondary displacement following conservative treatment in case 1 in mind follow-up CT scans of the cervical spine were performed four days after the initial injury revealing a mild secondary lateral displacement. Instability was confirmed fluoroscopically and posterior C1–C3/4 fusion was performed. Intraoperatively, the left posterior atlantoaxial membrane was found ruptured. The postoperative course was uneventful; CT scans confirmed an anatomic reduction and correct placement of all pedicular screws (Figure 2(b)). Being a French national the patient was repatriated one week postoperatively.

## 3. Discussion

Odontoid fractures account for approximately 10% of all cervical vertebrae fractures [2, 3]. In elderly patients with osteoporosis, these fractures may occur after minor trauma. Pepin et al. reported that 46% of all odontoid fractures occur in patients older than 60 years of age [9]. In recent years, in an aging society, the number of odontoid fractures is increasing [10–12].

The Anderson system is widely accepted for classification of odontoid fractures. Anderson and D'Alonzo reported that the prognosis of odontoid fractures depended on its fracture pattern. In this system, type I describes an oblique fracture of the tip of the dens, type II is a fracture at the junction of the dens and the central body of the axis, and type III is a fracture in which the fracture line extends downward into the cancellous portion of the body of the axis [1].

In case of treating type III fractures, the decision-making process is sometimes difficult. Optimal treatment for type III fracture is still controversially discussed [4–6]. Type III fracture line is in the body of the axis; thus, the contact area of the fracture is large. Additionally, the two main arteries supplying the odontoid are not likely to be disturbed anatomically [13]. For these reasons, conservative treatment with immobilization in a halo-vest or semirigid collars has traditionally been recommended for type III fractures [1–5]. Halo-vests may yield rigid fixation [4–6] but can be associated with emotional distress and potential morbidity, such as skin ulceration or pin dislocation [5, 6, 14]. Polin et al. reported that the Philadelphia collar was equivalent to the halo-vest in terms of clinical outcome [5]. According to the treatment algorithm for odontoid fractures suggested by Konieczny et al., the Philadelphia collar may be a good choice for stable type III fractures [6]. More unfavorable results have, however, been reported for conservative treatment in displaced fractures and doubt has been raised for elderly patients with osteoporosis [7, 9–12, 15].

The uncertainty about optimal treatment for type III fractures reflected in the available literature may partly be attributed to the fact that the definition given by Anderson and D'Alonzo was somewhat obscure. As a result, some axis body fractures may also be described as type III odontoid fractures. Benzel et al. pointed out that Anderson type III fractures do not genuinely involve the odontoid process and suggested that the application of the original classification of axis body fractures be more appropriate [16]. Nevertheless, this classification did not gain wide acceptance. To the authors best knowledge, only small case series exist on axis body fractures and conservative treatment was generally favored [17]. Some authors tried to recommend choice of treatment according to fracture pattern [18, 19].

In this series, we describe two cases of minimally displaced Anderson type III odontoid fractures with almost identical fracture patterns which we refer to as “oblique type axis body fractures.” The first case occurred following minor trauma in a frail patient. Conservative treatment with cervical spine immobilization was insufficient and resulted in gross displacement.

The second case was a result of a high-energy impact. The deleterious outcome of the first case led to a higher index of suspicion in the latter. As a consequence, the characteristic fracture pattern was identified and instability was detected early enough to allow for operative treatment performed before gross deformity could develop.

Several biomechanical studies compared the effectiveness of different surgical techniques for odontoid fractures [20, 21]. Anterior screw fixation is the method of choice in type II odontoid fractures and can also be performed in type III fractures. In fact, such an approach was considered by the authors for the patient described in case 2. Unfortunately, the severe obesity did not permit correct placement of the screws as soft tissue masses would prevent getting the desired angle as simulated on CT scans and during fluoroscopy imaging. Thus, posterior C1–C3/4 fixation was performed, despite the fact that C1-C2 posterior fusion will restrict atlantoaxial rotation by more than 50% [21]. Intraoperatively, we found the posterior atlantoaxial membrane ruptured on one side. It is reasonable to think that this ligamentous injury may have contributed to the instability. As we did not operate on the other patient it remains speculative whether a ligamentous injury was also present in that case. Whereas the low-energy trauma might be an argument against this theory the severe displacement could hardly have occurred without a concomitant ligamentous injury.

Very few comparable cases have been described in the past. Hähnle et al. reported three cases of shear fractures through the axis body, all of which were treated conservatively [22]. Patients were, however, substantially younger than in the present series. Goldschlager et al. reported one case of an oblique axis body fracture [23]. This patient was initially treated conservatively in a Philadelphia collar for three months but was converted to posterior C1–C3/4 fixation for nonunion. Mild, but no gross, displacement was reported and the authors did not describe ligamentous injuries.

In recent years, according to surgical advancements and an aging society, surgical treatment for type III odontoid fractures in elderly patients is becoming more common [10, 11, 15, 24]. Moreover, for early improvement of activities of daily living, aggressive surgical treatment may generally be a good choice for type III odontoid fractures [10, 11, 24, 25].

## 4. Conclusion

With regard to the present cases, we suspect that oblique type axis body fractures resemble a highly unstable subtype of Anderson type III fractures with the potential of severe secondary deformity following conservative treatment, irrespective of initial grade of displacement. The authors therefore warrant a high index of suspicion for this injury and suggest early operative stabilization. It is, however, important to state that only limited recommendations can be drawn from the few cases. Further studies are needed to confirm this theory and should focus on the influence of patient age, bone quality, concomitant ligamentous injury on the grade of instability, and clinical outcome.

## References

[1] Anderson L. D., D'Alonzo R. T. (1974). Fractures of the odontoid process of the axis. *The Journal of Bone & Joint Surgery—American Volume*.

[2] Hadley M. N., Browner C., Sonntag V. K. H. (1985). Axis fractures: a comprehensive review of management and treatment in 107 cases. *Neurosurgery*.

[3] Greene K. A., Dickman C. A., Marciano F. F., Drabier J. B., Hadley M. N., Sonntag V. K. H. (1997). Acute axis fractures. Analysis of management and outcome in 340 consecutive cases. *Spine*.

[4] Van Holsbeeck E., Stoffelen D., Fabry G. (1993). Fractures of the odontoid process. Conservative and operative treatment. Prognostic factors. *Acta Orthopaedica Belgica*.

[5] Polin R. S., Szabo T., Bogaev C. A., Replogle R. E., Jane J. A. (1996). Nonoperative management of Types II and III odontoid fractures: the Philadelphia collar versus the halo vest. *Neurosurgery*.

[6] Konieczny M. R., Gstrein A., Müller E. J. (2012). Treatment algorithm for dens fractures: non-halo immobilization, anterior screw fixation, or posterior transarticular C1–C2 fixation. *The Journal of Bone & Joint Surgery—American Volume*.

[7] Kirkpatrick J. S., Sheils T., Theiss S. M. (2004). Type-III dens fracture with distraction: an unstable injury—a report of three cases. *Journal of Bone and Joint Surgery Series: A*.

[8] Chiba K., Fujimura Y., Toyama Y., Fujii E., Nakanishi T., Hirabayashi K. (1996). Treatment protocol for fractures of the odontoid process. *Journal of Spinal Disorders*.

[9] Pepin J. W., Boume R. B., Hawkins R. J. (1985). Odontoid fractures, with special reference to the elderly patient. *Clinical Orthopaedics and Related Research*.

[10] Huybregts J. G. J., Jacobs W. C. H., Vleggeert-Lankamp C. L. A. M. (2013). The optimal treatment of type II and III odontoid fractures in the elderly: a systematic review. *European Spine Journal*.

[11] Platzer P., Thalhammer G., Oberleitner G., Schuster R., Vécsei V., Gaebler C. (2007). Surgical treatment of dens fractures in elderly patients. *The Journal of Bone & Joint Surgery—American Volume*.

[12] Wisoff H. S. (1984). Fracture of the dens in the aged. *Surgical Neurology*.

[13] Althoff B., Goldie I. F. (1977). The arterial supply of the odontoid process of the axis. *Acta Orthopaedica Scandinavica*.

[14] Horn E. M., Theodore N., Feiz-Erfan I., Lekovic G. P., Dickman C. A., Sonntag V. K. H. (2006). Complications of halo fixation in the elderly. *Journal of Neurosurgery: Spine*.

[15] Rao G., Apfelbaum R. I. (2005). Odontoid screw fixation for fresh and remote fractures. *Neurology India*.

[16] Benzel E. C., Hart B. L., Ball P. A., Baldwin N. G., Orrison W. W., Espinosa M. (1994). Fractures of the C-2 vertebral body. *Journal of Neurosurgery*.

[17] German J. W., Hart B. L., Benzel E. C. (2005). Nonoperative management of vertical C2 body fractures. *Neurosurgery*.

[18] Zhang Y. S., Zhang J. X., Yang Q. G., Shen C. L., Li W., Yin Z. S. (2014). Surgical management of the fractures of axis body: indications and surgical strategy. *European Spine Journal*.

[19] Fujimura Y., Nishi Y., Kobayashi K. (1996). Classification and treatment of axis body fractures. *Journal of Orthopaedic Trauma*.

[20] Richter M., Schmidt R., Claes L., Puhl W., Wilke H.-J. (2002). Posterior atlantoaxial fixation: biomechanical in vitro comparison of six different techniques. *Spine*.

[21] Grob D., Crisco J. J., Panjabi M. M., Wang P., Dvorak J. (1992). Biomechanical evaluation of four different posterior atlantoaxial fixation techniques. *Spine*.

[22] Hähnle U. R., Wisniewski T. F., Craig J. B. (1999). Shear fracture through the body of the axis vertebra. *Spine*.

[23] Goldschlager T., Leach J. C. D., Williamson O. D., Malham G. M. (2012). Oblique axis body fracture—pitfalls in management. *Injury*.

[24] Naderi S., Akşan Ö., Acar F., Mertol T., Arda N. (2006). Odontoid fractures. *Turkish Neurosurgery*.

[25] Hsu W. K., Anderson P. A. (2010). Odontoid fractures: update on management. *Journal of the American Academy of Orthopaedic Surgeons*.

